# Diversity of Hepatitis E Viruses in Rats in Yunnan Province and the Inner Mongolia Autonomous Region of China

**DOI:** 10.3390/v17040490

**Published:** 2025-03-28

**Authors:** Li-Li Li, Xiao-Hua Ma, Xiao-Wei Nan, Jing-Lin Wang, Jing Zhao, Xiao-Man Sun, Jin-Song Li, Gui-Sen Zheng, Zhao-Jun Duan

**Affiliations:** 1 National Key Laboratory of Intelligent Tracking and Forecasting for Infectious Diseases (NITFID), Beijing 102206, China; lill@ivdc.chinacdc.cn (L.-L.L.); sunxm@ivdc.chinacdc.cn (X.-M.S.); lijs@ivdc.chinacdc.cn (J.-S.L.); 2 NHC Key Laboratory for Medical Virology and Viral Diseases, Beijing 102206, China; 3 National Institute for Viral Disease Control and Prevention, China CDC, Beijing 102206, China; 13772775823@163.com; 4 GANSU Provincial Centers for Disease Control and Prevention, Lanzhou 730000, China; mxh0625@126.com; 5 Inner Mongolia Autonomous Region Center for Disease Control and Prevention (Inner Mongolia Autonomous Region Academy of Preventive Medicine), Hohhot 010080, China; nanxw0407@163.com; 6 Yunnan Tropical and Subtropical Animal Viral Disease Laboratory, Yunnan Animal Science and Veterinary Institute, Kunming 650224, China; wangjl107@163.com; 7 School of Public Health, Gansu University of Chinese Medicine, Lanzhou 730000, China

**Keywords:** rat hepatitis E virus (HEV), Hepeviridae, genetic diversity, complete genome, rodent

## Abstract

Hepatitis E virus (HEV) is one of the most common pathogens causing acute hepatitis. Rat HEV, a member of the genus *Rocahepevirus*, infects mainly rat but can also cause human zoonotic infection. A survey of the virome of rats via next-generation sequencing (NGS) was performed in Yunnan Province and Inner Mongolia in China. Further screening of rat HEV was conducted by nested PCR. The complete genome of six representative strains were obtained by NGS and RT-PCR. The virome analysis revealed that multiple reads were annotated as *Hepeviridae*. The screening results showed that HEV was detected in 9.6% (34 of 355) of the rat samples and phylogenetically classified into three lineages. The sequences from Yunnan clustered with *Rocahepevirus ratti*, named the YnRHEV group, and those from Inner Mongolia were separated into two lineages, named the NmRHEV-1 and NmRHEV-2 groups. Complete sequence analysis showed that YnRHEV had very high sequence identity to a human HEV strain identified in immunosuppressed patients (88.7% to 94.3%)*,* a reminder of the risk of cross-species transmission of rodent HEV. Notably, NmRHEV-1 and the most closely related rat HEV, RtCb-HEV/HeB2014, were divergent from other HEV. The phylogenetic analyses and lower sequence identities of the complete genome suggested the NmRHEV-1 to be a novel putative genus of the subfamily Orthohepevirinae. NmRHEV-2 shared the highest sequence identities (70.6% to 72.0%) with the species *Rocahepevirus eothenomi*, which may represent a putative novel genotype. This study revealed high genetic diversity of *Hepeviridae* in rats in China and a potentially zoonotic *Rocahepevirus ratti* strain.

## 1. Introduction

Hepatitis E virus (HEV) is a group of single-stranded, non-enveloped, positive-sense RNA viruses of the family Hepeviridae, subfamily Orthohepevirinae. The majority of HEV infections are asymptomatic, but some manifest as self-limiting acute hepatitis [1]. There are an estimated 20 million HEV infections annually worldwide, leading to about 60,000 fatalities [2,3]. Infection with HEV can cause acute liver failure and fetal loss and has a mortality rate of up to 25% in pregnant women [4]. The genome of HEV consists of three partially overlapping open reading frames (ORFs), a short 5′ untranslated region (UTR), and a 3′ UTR. ORF1 encodes a nonstructural polyprotein and ORF2 encodes a structural capsid protein. ORF3 overlaps partially with ORF2 and encodes a multifunctional phosphoprotein. Recently, a novel ORF4 overlapping the start of ORF1 was identified in strains belonging to the species *Orthohepevirus ratti* [5,6].

Based on the 2022 updated ICTV (International Committee on Taxonomy of Viruses) taxonomy, Hepeviridae are divided into two subfamilies (*Orthohepevirinae* and *Parahepevirinae*) [7]. HEV is a member of the Orthohepevirinae family, which contains four genera: Paslahepevirus, Avihepevirus, Rocahepevirus, and Chirohepevirus, corresponding to previous four genera (orthohepeviruses A, B, C, and D, respectively) [8,9].

*Paslahepevirus* is genetically diverse with at least eight genotypes (1 to 8) and infects mainly humans but has a broad range of hosts [10,11]. Genotypes 1 and 2 are limited to humans, and genotypes 3, 4, and 7 cause zoonotic infections of human and animals; the other genotypes infect mainly animals [3]. Rodents are the major hosts of *Rocahepevirus*, but the recent identification of *Rocahepevirus* from several animal species (shrew, voles, brown bear, ferret, mink kestrel/falcon, and red fox) indicate it to have a broad host range and high genetic diversity [3,12,13]. *Rocahepevirus* comprises two species (*Rocahepevirus ratti* and *Rocahepevirus eothenomi*); Based on updated nomenclature, previous genotypes HEV-C1 and HEV-C2 are now classified into *Rocahepevirus ratti*, and putative genotype HEV-C4 is classified into *Rocahepevirus eothenomi*. HEV-C3 and HEV-C4 are novel putative genotypes reported in Chevrier’s field mouse and Père David’s vole in 2018 [1]. Thus far, *Rocahepevirus* sequences have been detected in 22 countries and 25 animal species, containing 8 species within the family Muridae and 10 species within the family Cricetidae of the order Rodentia [14]. *Avihepevirus* has been identified only in avians and *Chirohepevirus* only in bats [1,3,15].

Rodents, one of the largest orders of mammals, are the main hosts of *Rocahepevirus* [16]. *Rocahepevirus* was first identified in the feces of a Norwegian rat in Germany in 2010 [17] and has subsequently been reported worldwide in rats [9,18,19,20,21,22]. *Rocahepevirus* was initially not considered to have zoonotic potential based on its divergence from *Paslahepevirus*, with which it has 50% to 60% nucleotide sequence identities to *Paslahepevirus* [23]. However, rat-derived *Rocahepevirus* is considered as zoonotic because some strains can cause acute or chronic symptomatic hepatitis in patients and immunosuppressed individuals in recent reports [24,25,26,27,28]. A human hepatitis E surveillance system has been established for several years in China. It mainly focuses on human HEV-A variants.

Yunnan Province and the Inner Mongolia Autonomous Region are located in southern and northern China, respectively. Both areas have high species diversity and a large number of rodents. In this study, we investigated the prevalence and genomic characteristics of HEVs in rodent animals. We identified several potentially zoonotic *Rocahepevirus* strains in Yunnan Province and a putative novel Hepeviridae genus and a putative novel *Rocahepevirus* genotype in the Inner Mongolia Autonomous Region, highlighting the diversity of rat HEV and their potential threat to public health.

## 2. Materials and Methods

### 2.1. Sample Collection and Storage

Between June 2016 and October 2017, 255 samples of liver tissue and intestinal contents from rodents trapped in the Inner Mongolia Autonomous Region (IMAR) were collected by local CDC. In addition, 100 liver samples of rodents in Yunnan Province (YN) were collected by the Yunnan Animal Science and Veterinary Institute. All animals were trapped in the field and biopsied in a Biosafety Level II laboratory. To avoid cross-contamination among samples, the anatomical tools were autoclaved between uses. The samples were transported to the National Institute for Viral Disease Control and Prevention using approved institutional biosafety protocols and stored at −80 °C until further analysis.

### 2.2. Sample Pretreatment

Samples were homogenized in minimum essential medium (MEM) and centrifuged to obtain supernatants. For next-generation sequencing (NGS), supernatants were passed through 0.22 μm filters to remove cell debris, and treated with OmniCleave Endonuclease (Epicenter, Madison, WI, USA) to digest unprotected nucleic acids. Total nucleic acids were extracted using the QIAamp MinElute Virus Spin Kit (Qiagen, Hilden, Germany).

### 2.3. NGS and Full-Length Genome Sequencing of HEV

The 355 rat samples were subjected to NGS. For sequencing, 15 to 25 samples were pooled according to tissue type, resulting in 21 pools. Library construction and sequencing were performed by Magigene Biotechnology Co., Ltd. (Guangzhou, China). Briefly, total nucleic acids were amplified using the REPLI-g Cell WGA&WTA Kit (Qiagen). Next, libraries were constructed using the NEBNext ^®^Ultra II library DNA Library Prep Kit for Illumina (NEB, Ipswich, MA, USA) and sequencing was performed using the Illumina Novaseq 6000 system (PE150 model). Bioinformatics analysis was carried out using our lab bioinformatics analysis platform [29]. To get complete genome of HEV, six single HEV-positive sample were selected for NGS based on the sequence heterogeneity of sanger sequence below mentioned. The assembled sequences of NGS were confirmed by RT-PCR and the 5′- and 3′- ends were amplified using the SMARTer RACE 5′/3′ Kit (Clotech, Escondido, CA, USA).

### 2.4. Detection of HEV by Nested Broad-Spectrum PCR and Host Species Identification

Nested broad-spectrum RT-PCR targeting 334 bp conserved region of the RNA-dependent RNA polymerase (*RdRp*) gene was performed to screen for HEV in rodent animals and for sanger sequencing [30]. Rat species were identified by amplifying a 248-bp fragment of mitochondrial cytochrome (*CytB*) gene as described previously [31].

### 2.5. Phylogenetic and Sequence Analyses

HEV-related reference sequences were downloaded from GenBank. Sequence alignment was performed using MEGA(version 10.1). Maximum-likelihood phylogenetic trees were constructed using MEGA-X with the general time reversible nucleotide substitution model; the bootstrap value was set to 1000. Sequence identities were calculated using the MegaAlign tool in DNAStar software(version 15).

## 3. Results

### 3.1. Overview of NGS Data

Twenty-one pools were generated for NGS, yielding 70 to 80 million reads per pool. Bioinformatics analysis revealed 24 Hepeviridae-related contigs in eight pools of 1500 to 6927 bp in length. The nucleotide identities among these contigs were between 48.6% to 99.3%. By comparison with known HEVs, the nucleotide acid identities ranged from 46.3% to 97.2%, suggesting the presence of genetic diversity and even novel HEV in these rodent animals. We next used nested broad-spectrum RT-PCR to screen the HEV for all 355 rats.

### 3.2. Prevalence and Phylogenetic Analysis of Hepeviridae in Rat

**Figure 1:**
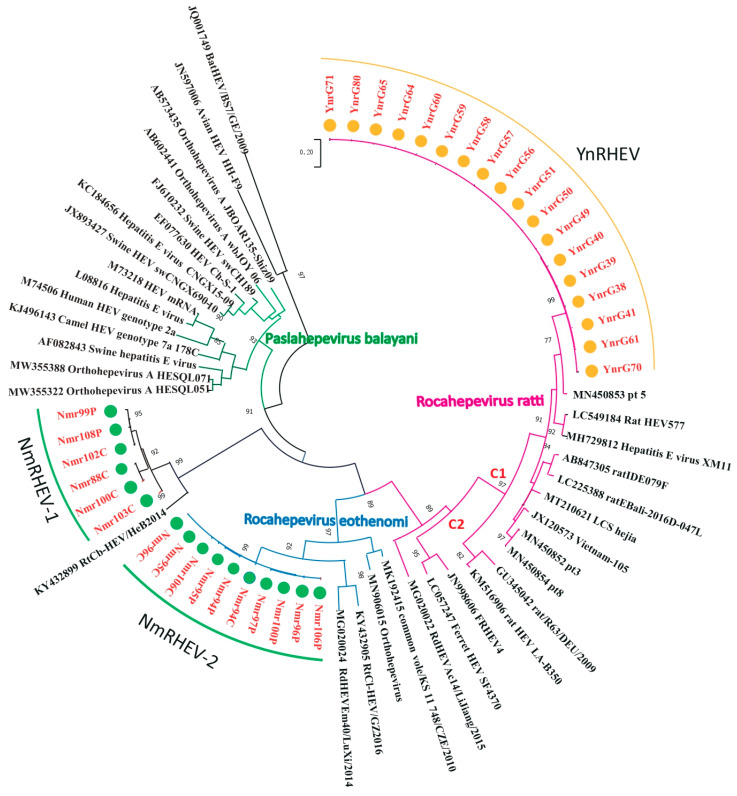
Maximum-likelihood phylogenetic tree based on the HEV partial *RdRp* gene sequence (334 nt). Bootstrap values were calculated after 1000 replicates. Only bootstrap values > 70% are shown; strains identified in this study are marked by yellow (YN) and green (IMAR) dots.

Nested broad-spectrum RT-PCR resulted in the detection of HEV in 34 of 355 (9.6%) rats, with 18 (18.0%) samples from YN and 16 (6.3%) from the IMAR. Phylogenetic analysis revealed that all the HEV sequences clustered into three lineages (Figure 1), suggesting high genetic diversity. The 18 sequences from YN shared 98.2% to 100% nucleotide sequence identities and all clustered into the *Rocahepevirus ratti* genotype C1 clade (Figure 1); on this basis, they were named the YnRHEV group. These sequences from YN shared 89% to 94.2% nucleotide sequence identities with known HEVs and all originated from *Rattus norvegicus*. The 16 sequences from the IMAR were classified phylogenetically into two clusters; one clustered as an outgroup with the genus *Rocahepevirus* lineage but distant from *Rocahepevirus ratti* genotype C1 to C4, and the other clustered as a outgroup with species *Rocahepevirus eothenomi* (HEV-C4 genotype) (Figure 1); they were named the NmRHEV-1 and NmRHEV-2 groups, respectively. NmRHEV-1 had the highest nucleotide sequence identities (73.1% to 74.9%) with RtCb-HEV/HeB2014 and was identified in *Mus musculus*, *Cricetulus sokolovi*, *Apodemus agrarius*, and *Cricetulus barabensis* (Table 1). NmRHEV-2 shared 70.6% to 72.0% nucleotide sequence identities with members of species *Rocahepevirus eothenomi* and was identified only in *Microtus fortis* and *Cricetulus barabensis* (Table 1).

### 3.3. Characteristics of the HEV Genome

Based on NGS data and phylogenetic analysis of a portion of the *RdRp* gene above, we selected six samples for complete genome sequencing by single-sample NGS combined with PCR and RACE. In agreement with the screening results, six strains were phylogenetically classified into the groups YnRHEV (YnrG58, YnrG38, and YnrG41), NmRHEV-1 (Nmr103c and Nmr99p), and NmRHEV-2 (Nmr94c).

The complete genomes (excluding the poly (A)-tail) comprised 7002 nt for YnRHEV, 7219 nt for NmRHEV-1, and 6943 nt for NmRHEV-2. The G+C content (excluding the poly (A)-tail) was 56.8% to 57.3% for YnRHEV and 49.3% for NmRHEV-2. The G+C content of NmRHEV-1 was 45.6% and 45.9%, which was lower than that of any other members of the family Hepeviridae (50.6% to 58.7%), even including the subfamily *Parahepevirinae* (49.68% to 50.56%).

**Figure 2:**
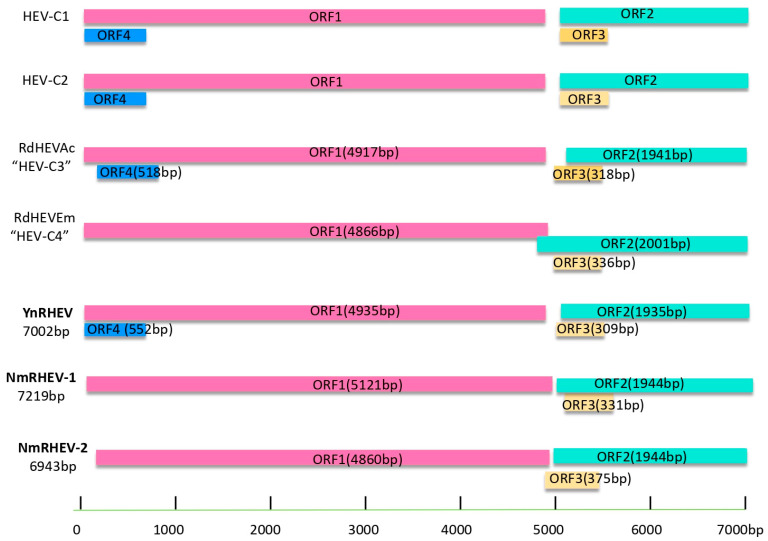
Schematic description of the species *Rocahepevirus* genomes and viral proteins.

The three groups had three typical HEV ORFs (ORF1 to ORF3) encoding, in order, a non-structural polyprotein, capsid protein, and a small phosphoprotein. YnRHEV also had a putative ORF4 overlapping with ORF1 (Figure 2). All sequences were deposited into GenBank under accession numbers MZ868954, MZ542728, MZ643466, ON009840, ON009841, and PV126640.

### 3.4. Comparative Analysis of Complete Genome Sequences

The nucleotide sequence identities among the three groups were 47.7% to 50.5%. The intra-group nucleotide sequence identities were 89.9% and 94.7% for the YnRHEV and NmRHEV-1 groups, respectively. Sequence comparison showed that the YnRHEV group was most closely related to *Rocahepevirus ratti* genotype C1, with 88.9% to 94.3% sequence identities. YnRHEV had the highest sequence identity with a human HEV strain, pt 5, identified in a kidney-transplant patient in Hong Kong in 2021 [27], with 94.3% (YnrG41) and 88.7% (YnrG38 and YnrG58) identities, respectively (Table 1). Notably, YnrG41 shared up to 98.0% and 97.5% amino acid sequence identities with pt 5 in the ORF1 and ORF2 regions. Strain pt 5 was formerly most closely related to the rat *Rocahepevirus ratti* genotype C1 strain ESUMBAWA-140L (85.2% nucleotide sequence identity).

The sequence analysis suggested that the NmRHEV-1 group represents a new lineage divergent from any known species. NmRHEV-1 was genetically similar to RtCb-HEV/HeB2014, a strain discovered in striped dwarf hamster in China (~77% nt identity), but this strain did not have a definitive classification. However, NmRHEV-1 and RtCb-HEV/HeB2014 were divergent from most other known viruses (43.4% to 53.5% nt identities). NmRHEV-1 had the highest nucleotide sequence identities with *Paslahepevirus* and *Rocahepevirus* (52.8% to 53.5% and 50.3% to 52.6%, respectively) and was divergent from species of *Avihepevirus* and *Chirohepevirus* (<50% nucleotide sequence identities). Similar results were obtained for the amino acid sequence identities in the ORF1 to ORF2 regions (Table 2). These results indicate the members of the NmRHEV-1 group to be novel viruses.

Sequence alignment suggested that NmRHEV-2 was divergent from known HEVs. NmRHEV-2 was most related to *Rocahepevirus eothenomi*, previously HEV-C4 (68.6% nucleotide sequence identity), suggesting that NmRHEV-2 is a novel genotype of *Rocahepevirus Eothenomi*, tentatively named HEV-C5.

### 3.5. Phylogenetic Analysis of Complete Genomes

**Figure 3:**
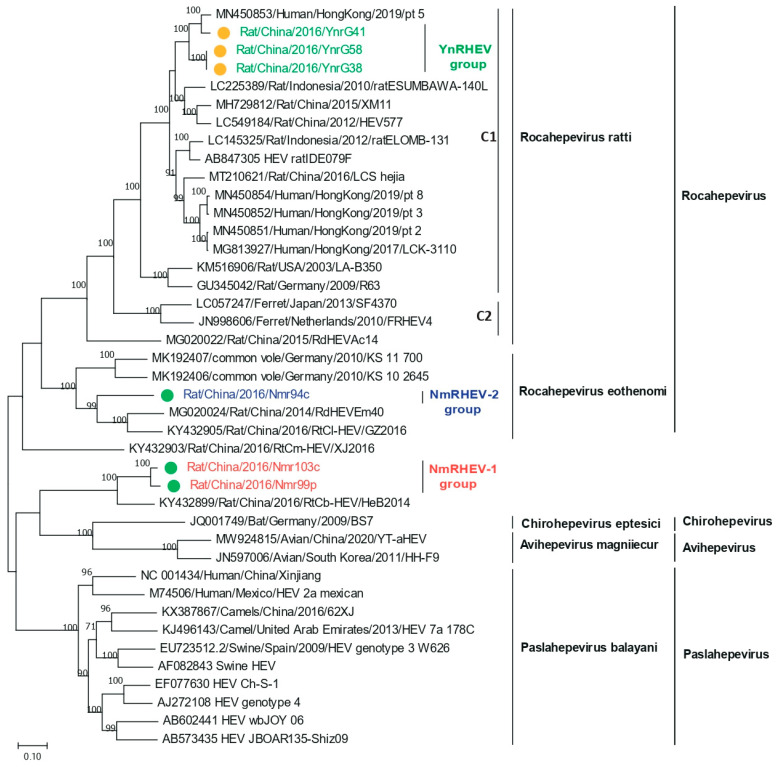
Phylogenetic analysis of complete or nearly complete nucleotide sequences of HEVs identified in this study and related HEV strains. Green and yellow dots indicate strains from Inner Mongolia and Yunnan Province, respectively.

ML trees were constructed based on the HEV nucleotide and amino acid sequences. Consistent with the screening results, the six complete genomes were separated into three groups based on the phylogenetic analysis. The YnRHEV group clustered with the *Rocahepevirus ratti* lineage, and strain YnrG41 clustered with pt 5 and with YnrG38 and YnrG58 (Figure 3). YnRHEV was phylogenetically closer to pt 5 than rat ESUMBAWA-140L, the strain most closely related to pt 5 at the time of its discovery, suggesting a possibility of cross-species transmission from rat to human.

**Figure 4:**
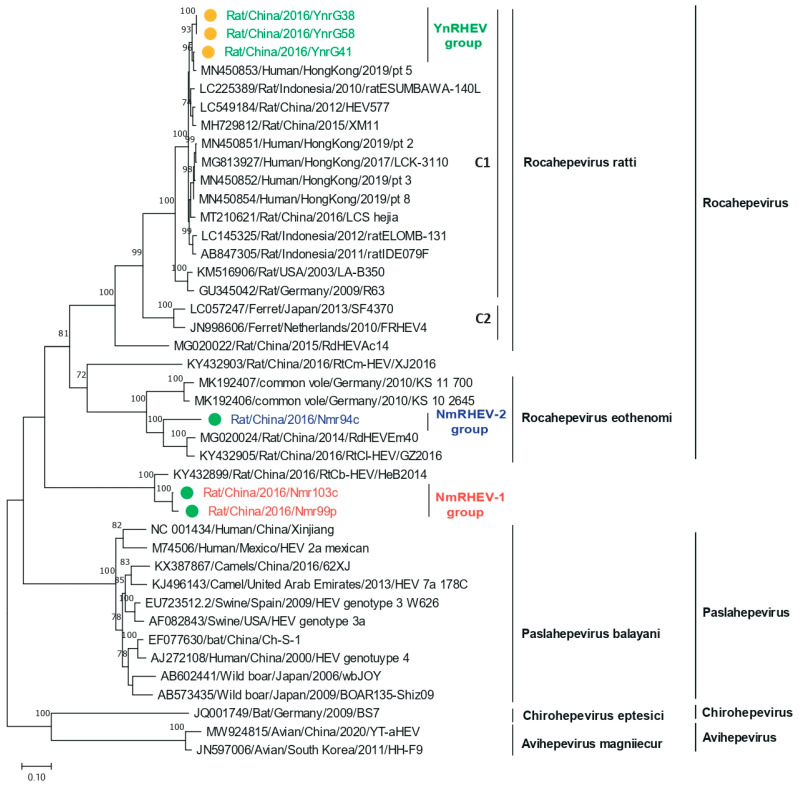
Phylogenetic analysis of the ORF1 amino acid sequences of HEVs identified in this study and of related HEV strains. Green and yellow dots indicate strains from Inner Mongolia and Yunnan Province, respectively.

**Figure 5:**
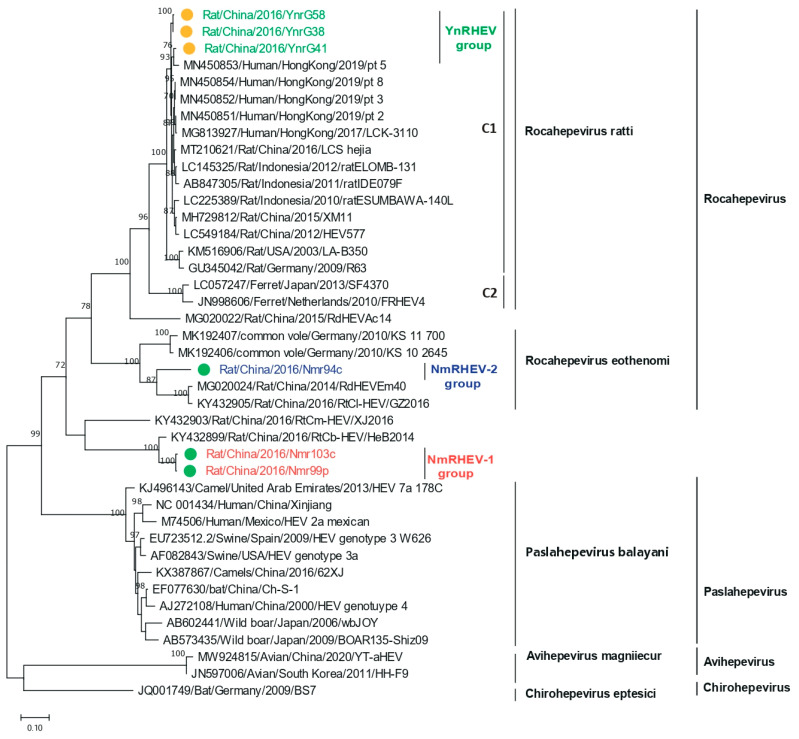
Phylogenetic analysis of the ORF2 amino acid sequences of HEVs identified in this study and of related HEV strains. Green and yellow dots indicate strains from Inner Mongolia and Yunnan Province, respectively.

The nucleotide acid tree of complete genome showed that NmRHEV-1 clustered with the rat strain RtCb-HEV/HeB2014, and NmRHEV-1 and RtCb-HEV/HeB2014 formed a separate monophyletic branch as an outgroup of the genera *Chirohepevirus* and *Avihepevirus* (Figure 3). However, in the ORF1 and ORF2 amino acid tree, NmRHEV-1 was classified as an outgroup to the clades of the genus *Rocahepevirus (*Figure 4 and Figure 5). Different topologies in the different region phylogenetic tree are common for distantly related species. For some strains, it may be caused by recombination; however, we did not found any recombination signal for NmRHEV-1. Here, the different topological position of NmRHEV-1 may be caused by the nucleotide acid or amino acid used for constructing the phylogenetic tree or caused by a more distanced relationship between NmRHEV-1 and known HEVs in terms of evolution position. In addition, the phylogenetic tree showed that NmRHEV-2 was clustered with members of *Rocahepevirus Eothenomi* in three phylogenetic trees, supporting NmRHEV-2 as a member of *Rocahepevirus Eothenomi*.

The lower sequence identities and the divergent phylogenetic topology from other HEV sequences suggest NmRHEV-1 to be a novel virus. The four Hepeviridae genera were phylogenetically distinct based on analysis of ORF1 codon positions 1 to 450 (methyltransferase), ORF1 codon positions 971 to 1692 (*RdRp* protein), and ORF2 codon positions 121 to 473 (capsid protein), so we constructed trees for these three regions. The methyltransferase and *RdRp* trees revealed that NmRHEV-1 and RtCb-HEV/HeB2014 are outgroups of *Chirohepevirus* and *Avihepevirus* (Figures S1 and S2), and in the ORF2 codon positions 121 to 473 tree, NmRHEV-1 clustered with *Rocahepevirus eothenomi* (Figure S3). The topological positions of NmRHEV-1 and RtCb-HEV/HeB2014 in the phylogenetic trees and extremely low identities with other HEV suggest NmRHEV-1 and RtCb-HEV/HeB2014 to be members of a novel genus of the family Hepeviridae.

NmRHEV-2 was phylogenetically classified into the species *Rocahepevirus eothenomi*; the sequence identities and phylogenetic analysis supported NmRHEV-2 as a novel genotype within *Rocahepevirus eothenomi*. Voles are the hosts of *Rocahepevirus eothenomi*; similarly, in our study, the hosts of NmRHEV-2 were *Microtus fortis* (a species of vole) and *Cricetulus barabensis* (a hamster). This indicates that *Rocahepevirus eothenomi* has a broad host range. In addition, NmRHEV-2 clustered with strains RdEm40 and GZ2016 (both from China).

## 4. Discussion

In this study, we detected human infection-related rat HEV strains in rat samples, indicating that these viruses circulate in rat populations. The HEV sequences were classified phylogenetically into three lineages, which shared only ~50% sequence identities, suggesting high genetic diversity of HEV in China. Moreover, we identified two potential novel rat HEVs, divergent from known HEVs, that may be members of a novel genus of Hepeviridae and a novel genotype within the species *Rocahepevirus eothenomi*.

HEVs with zoonotic characteristics have high genetic diversity and a broad host range [6,32,33]. The major zoonotic *Paslahepevirus* subtypes are HEV-A3 and HEV-A4 [3]. Notably, some members of *Rocahepevirus ratti* cause zoonotic infections in immunocompromised individuals and acute HEV-infected patients and have emerged in Hong Kong, central Africa, and Spain [24,25,27,28,34]. In 2021, Yuen detected a rat HEV in patients with hepatitis (*n* = 6 of 2201) and in immunocompromised individuals (n = 1 of 659). Strain pt 5 was identified in a patient with persistent hepatitis who had undergone kidney transplant and was most closely related to the rat HEV ratESUMBAWA-140L (84.5% nucleotide sequence identity) [27]. In this study, the YnRHEV group was closely related to pt 5 (94.3% nucleotide and >97% aa sequence identities in ORF1 and ORF2). Phylogenetic analysis suggested a closer relationship between YnRHEV and pt 5, suggesting that YnRHEVs have a common close ancestor with the strains that have the potential to infect humans. Four *Rocahepevirus* lineages capable of infecting humans have been identified in Canada, Hong Kong, and Spain [27,35]; our findings provided further evidence that some *Rocahepevirus ratti* strains are capable of cross-species transmission. The high prevalence of the virus and the frequent contact between humans and rats increase the likelihood of transmission of *Rocahepevirus ratti* [12,25,36].

The human hepatitis E surveillance system in China is mainly focused on human HEV-A. Most commercial molecular test methods targeting HEV-A variants cannot detect *Rocahepevirus ratti* genotype C1 RNA due to low nucleotide identity. However, some serological assays for HEV may be sensitive to this genetic variant. In a previous study, a monoclonal antibody raised against the HEV-3 capsid protein showed cross-reactivity with capsid protein derivatives of several genotypes of Paslahepevirus balayani and *Orthohepevirus ratti*. Another study demonstrated that some commercial HEV-A antibody test kits are sensitive to *Orthohepevirus ratti* genotype C1 (Sridhar et al., 2021 [24]). These findings suggest that true *Orthohepevirus ratti* genotype C1 may be misidentified as HEV-A infection if a specific nucleic acid test is lacking. Consequently, infection with this genetic variant could be missed by both molecular and serological tests.

Current HEV-A serological diagnostic kits should be evaluated for their sensitivity to Rocahepevirus ratti genotype C1. Additionally, a specific nucleic acid test method for this genetic strain should be established or assessed for further use. The surveillance of circulating HEV strains in human and rodent species would provide insight into the risk of cross-species transmission of rodent HEV.

Several *Rocahepevirus*-related viruses have been identified [1]. In this study, we identified two novel groups of Hepeviridae, divergent from known HEVs. NmRHEV-1 was divergent from known HEV strains (<55% identity) apart from the unclassified RtCb-HEV/HeB2014. NmRHEV-1 and RtCb-HEV/HeB2014 formed a monophyletic clade outside the *Paslahepevirus*, *Rocahepevirus*, *Chirohepevirus*, and *Avihepevirus* lineages. Based on their phylogenetic classifications, NmRHEV-1 and RtCb-HEV/HeB2014 may be members of a novel genus of Hepeviridae. NmRHEV-1 had a G+C content of ~45%, lower than that of other *Hepeviridae* (50.64% to 57.33%)*,* supporting the classification of NmRHEV-1 as a novel genus.

There is, at present, no monitoring of rat HEV in China. Prior reports on rat HEV in China focused on their prevalence and diversity. Surveillance of rat HEV is focused on southern China; none has been reported in northern China. In this study, rat HEVs were detected in 18.0% and 6.3% of samples from Yunnan Province and Inner Mongolia Autonomous Region, respectively. The previous study revealed that the prevalence was 20.2% to 57.9% in several regions of southern China [12,37,38]. The prevalence of rat HEV is higher in southern than northern China, and its genetic diversity is higher in northern China. The prevalence of strains differed in northern and southern China, possibly because of geographical isolation of rats in southern and northern China. Globally, the prevalence of rat HEV is 5.6% in Canada [39] and 1.2% in Japan [40], and the average detection rates were 35.3%, 11.5%, 2.9%, and 7.1% in the German cities of Hamburg, Berlin, Stuttgart, and Esslingen, respectively [26]. Rat HEV was detected in rats in 11 European countries, with an average detection rate of 12.4% [22]. Therefore, *Rocahepevirus* has a broad geographical distribution and a high prevalence worldwide.

## 5. Conclusions

Human HEV infections can be caused not only by *Paslahepevirus* but also by members of the genus *Rocahepevirus ratti*, which is likely to be missed by molecular diagnostics for hepatitis. Because of the risk of zoonotic infection, screening for *Rocahepevirus ratti* should be performed in patients with hepatitis. We identified two novel members of the Hepeviridae, which expands the genetic diversity of Hepeviridae. Enhanced surveillance is needed to provide insight into the prevalence and genetic characteristics of rat HEVs.

## Figures and Tables

**Table 1:** The information of HEV-positive samples.

Group	Sample ID	Site	Date	Host Species	Virus Classification
YnRHEV	16Ynr-38	YN	June 2016	*Rattus norvegicus*	*Rocahepevirus ratti*
	16Ynr-39	YN	June 2016	*Rattus norvegicus*	*Rocahepevirus ratti*
	16Ynr-40	YN	June 2016	*Rattus norvegicus*	*Rocahepevirus ratti*
	16Ynr-41	YN	June 2016	*Rattus norvegicus*	*Rocahepevirus ratti*
	16Ynr-49	YN	September 2016	*Rattus norvegicus*	*Rocahepevirus ratti*
	16Ynr-50	YN	September 2016	*Rattus norvegicus*	*Rocahepevirus ratti*
	16Ynr-51	YN	September 2016	*Rattus norvegicus*	*Rocahepevirus ratti*
	16Ynr-56	YN	September 2016	*Rattus norvegicus*	*Rocahepevirus ratti*
	16Ynr-57	YN	September 2016	*Rattus norvegicus*	*Rocahepevirus ratti*
	16Ynr-58	YN	September 2016	*Rattus norvegicus*	*Rocahepevirus ratti*
	16Ynr-59	YN	September 2016	*Rattus norvegicus*	*Rocahepevirus ratti*
	16Ynr-60	YN	September 2016	*Rattus norvegicus*	*Rocahepevirus ratti*
	16Ynr-61	YN	October 2013	*Rattus norvegicus*	*Rocahepevirus ratti*
	16Ynr-64	YN	November 2016	*Rattus norvegicus*	*Rocahepevirus ratti*
	16Ynr-65	YN	November 2016	*Rattus norvegicus*	*Rocahepevirus ratti*
	16Ynr-70	YN	November 2016	*Rattus norvegicus*	*Rocahepevirus ratti*
	16Ynr-71	YN	November 2016	*Rattus norvegicus*	*Rocahepevirus ratti*
	16Ynr-80	YN	November 2016	*Rattus norvegicus*	*Rocahepevirus ratti*
NmRHEV-1	17Nmr-103C	IMAR	October 2017	*Apodemus agrarius*	Novel orthohepeviruses
	17Nmr-99P	IMAR	June 2017	*Cricetulus sokolovi*	Novel orthohepeviruses
	17Nmr-100C	IMAR	October 2017	*Cricetulus barabensis*	Novel orthohepeviruses
	17Nmr-88C	IMAR	June 2017	*Mus musculus*	Novel orthohepeviruses
	17Nmr-102C	IMAR	October 2017	*Cricetulus barabensis*	Novel orthohepeviruses
	17Nmr-108P	IMAR	October 2017	unidentified	Novel orthohepeviruses
NmRHEV-2	17Nmr-96C	IMAR	June 2017	*Microtus fortis*	*Rocahepevirus Eothenomi*
	17Nmr-95C	IMAR	June 2017	*Microtus fortis*	*Rocahepevirus Eothenomi*
	17Nmr-95P	IMAR	June 2017	*Microtus fortis*	*Rocahepevirus Eothenomi*
	17Nmr-94P	IMAR	June 2017	*Microtus fortis*	*Rocahepevirus Eothenomi*
	17Nmr-106C	IMAR	October 2017	*Microtus fortis*	*Rocahepevirus Eothenomi*
	17Nmr-94C	IMAR	June 2017	*Microtus fortis*	*Rocahepevirus Eothenomi*
	17Nmr-97P	IMAR	June 2017	*Microtus fortis*	*Rocahepevirus Eothenomi*
	17Nmr-100P	IMAR	October 2017	*Cricetulus barabensis*	*Rocahepevirus Eothenomi*
	17Nmr-96P	IMAR	June 2017	*Microtus fortis*	*Rocahepevirus Eothenomi*
	17Nmr-106P	IMAR	October 2017	*Microtus fortis*	*Rocahepevirus Eothenomi*

**Table 2:** Sequence identities of strains identified in this study with reference HEVs.

	Nucleotide Sequences (%)				Amino Acid Sequences (%)		
Genotype/Strain	Complete	ORF1	ORF2	ORF3	ORF1	ORF2	ORF3
YnRHEV							
YnrG38/G58/G41	89.3–99.0	88.8–99.0	84.6–98.1	93.8–100	92.6–96.3	97.6	94.5
NmRHEV-1	50.2–50.5	45.5–48.5	57.2–57.6	46.4–48.8	48.9–51.6	56.7–57.3	45–46.1
NmRHEV-2	54.6–56.7	48.9–49.0	58.9–62.1	48.9–51.2	54.6–56.7	57.3–59.2	45.2–65.3
HEV-C1	88.9–94.3	88.6–94.3	90.1–94.4	94.4–97.4	90.1–98.0	96.8–97.5	94.9
HEV-C2	63.2–63.6	64.5–64.8	71.9–72	58.5–59.7	73.6–77.4	80.4–80.7	62.5–63.4
HEV-C3	64.5–64.8	61.3–61.8	68.1–68.2	58.7	67.6–70.5	74.2	59.0–59.6
HEV-C4	61.3–61.8	50.1–50.2	61.5–62.0	39.7–39.8	54.5–56.8	61.5–61.8	41.6–43.1
HEV-A	52.1–53.0	49.4–50.8	56.1–56.4	47.9–51.3	48.4–51.4	55.8–56.6	20.1–21.4
HEV-B	45.8–46.4	45.9–46.1	47.2–47.3	34.1–35.0	47.1–49.4	43.4–44.0	25.9–26.3
HEV-D	46.5–46.8	45.0–45.1	49.9–50.3	35.6–36.2	47.6–49.3	48.8–49.1	11.8–13.1
NmRHEV-1							
Nmr103c/Nmr99p	95.4	94.8	96.9	98.5	97.8	99.2	98.1
NmRHEV-2	51.8–52.3	47.7–47.9	58.1–59.2	52.1–52.9	51.8–52.3	56.2–58.3	48.2–49.5
RtCb-HEV/HeB2014	77.6–77.7	75.5–75.8	83.2–83.6	83.9–84.6	87.5–88.5	91.3–91.4	92.3
HEV-C1	50.3–50.6	48.1–48.2	56.5–56.8	43.1–43.8	48.3–49.0	56.6–56.7	36.3
HEV-C2	51.3–51.3	47.3–47.5	56.5–57	37.2–37.6	48.7–49.2	57.1–57.2	34.1
HEV-C3	51.7	47.8	56.3–56.4	42.9–43.6	48–48.5	55.0–55.1	32.6
HEV-C4	52.6	54.2–54.4	58.1–58.3	37.6–38.1	47.6–48.5	59.1–59.3	26.5
HEV-A	52.8–53.5	52.5–53.4	53.9–54.9	35.6–38.2	44.3–49.2	54.6–55.7	21.5–24.4
HEV-B	43.4–43.7	42.4–42.6	46.3–47.0	27.9–38.3	41.9–42.1	42.2–42.3	22.2
HEV-D	46.1	44.9–45.0	48.9–49.0	30.2–30.6	41.4–41.8	49.2–49.5	21.5

## Data Availability

The nucleotide sequences in this study are derived from or deposited to the NCBI GenBank database (https://www.ncbi.nlm.nih.gov).

## Supplementary Materials

The following supporting information can be downloaded at: https://www.mdpi.com/article/10.3390/v17040490/s1, Figure S1: Phylogenetic analysis of ORF1 codon positions 1 to 450 (methyltransferase) of HEVs identified in this study and related strains; Figure S2: Phylogenetic analysis of ORF1 codon positions 971 to 1692 (RdRp) of HEVs identified in this study and related HEV strains; Figure S3: Phylogenetic analysis of ORF2 codon positions 121 to 473 (capsid protein) of HEVs identified in this study and related HEV strains.
