# Supplementary material for: Diversity of Hepatitis E Viruses in Rats in Yunnan Province and the Inner Mongolia Autonomous Region of China
Source: Viruses. 2025 Mar 28;17(4):490. doi: 10.3390/v17040490 (PMC12031282; doi:10.3390/v17040490)
Supplement: Supplementary file 1 [file viruses-17-00490-s001.zip › viruses-3397592-supplementary.pdf]

SUPPLEMENTARY FIGURES

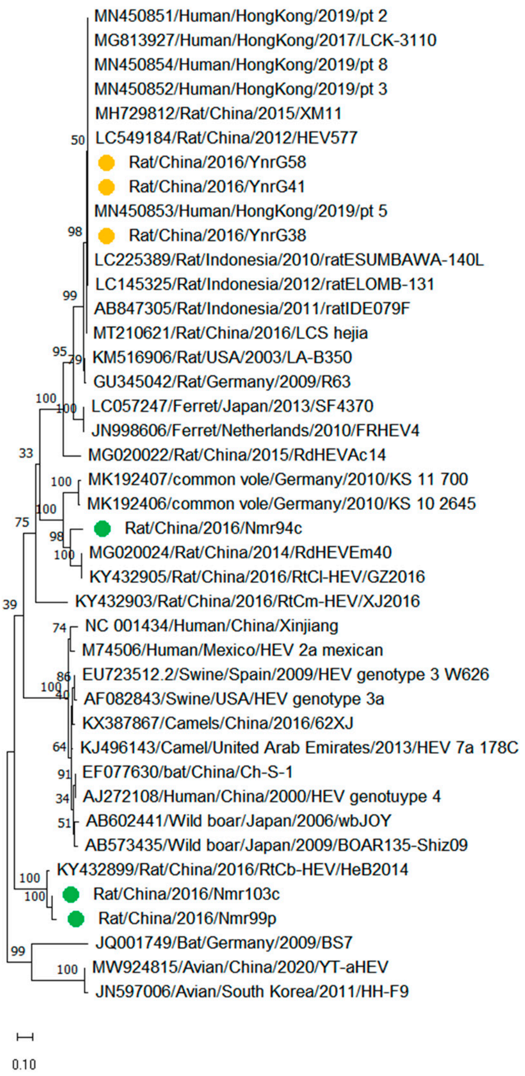

Figure S1. Phylogenetic analysis of ORF1 codon positions 1 to 450 (methyltransferase) of HEVs identified in this study and related strains. Green and yellow dots were strains from Inner Mongolia and Yunnan Province, respectively.

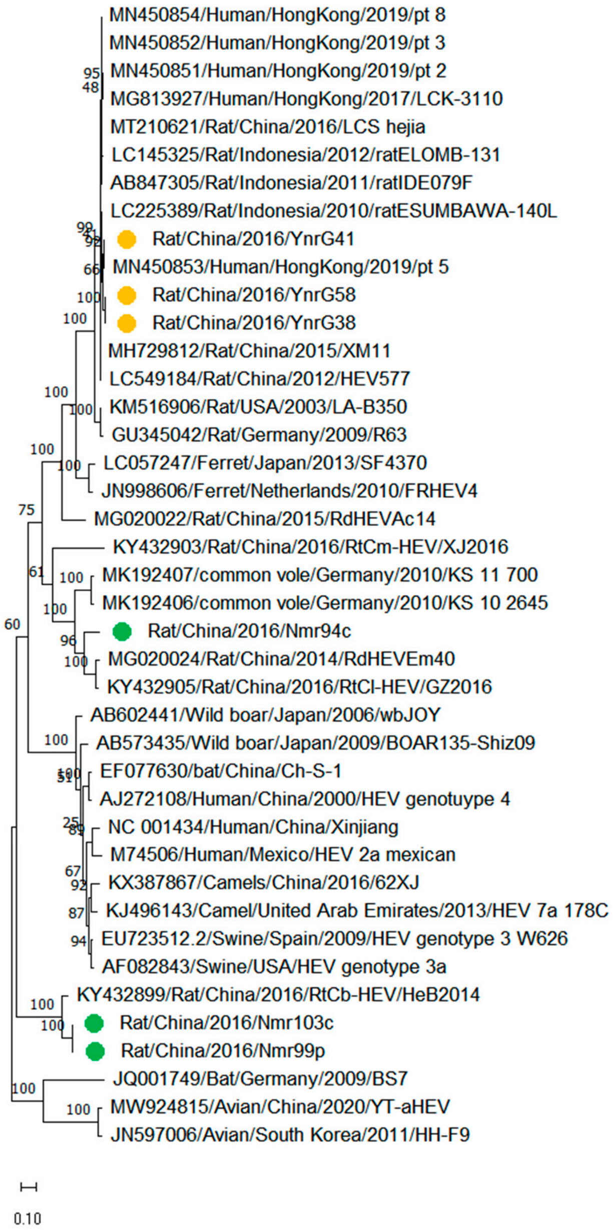

Figure S2. Phylogenetic analysis of ORF1 codon positions 971 to 1692 (RdRp) of HEVs identified in this study and related HEV strains. Green and yellow dots were the strains from Inner Mongolia and Yunnan Province, respectively.

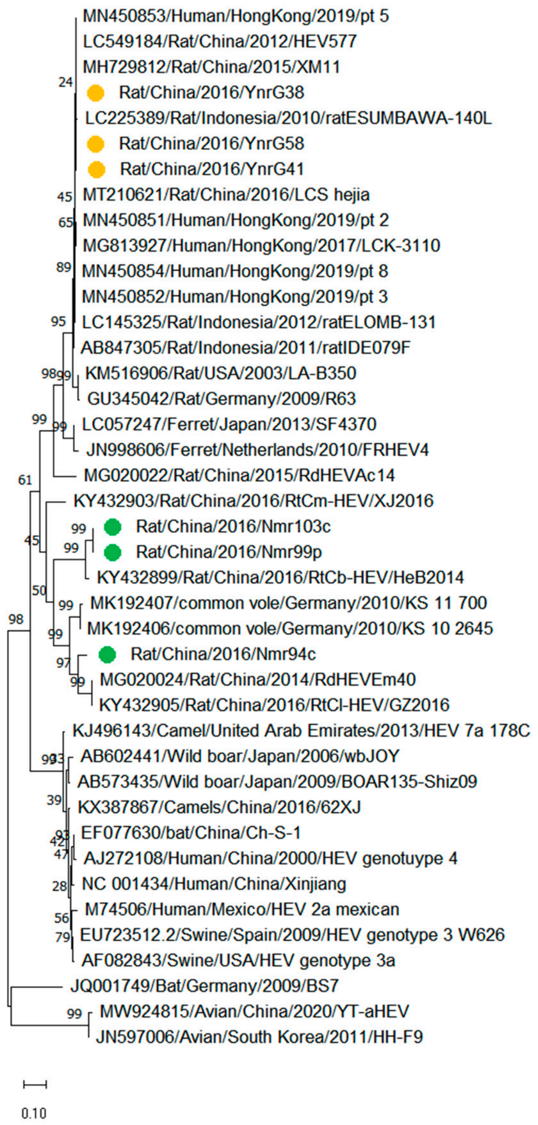

Figure S3. Phylogenetic analysis of ORF2 codon positions 121 to 473 (capsid protein) of HEVs identified in this study and related HEV strains. Green and yellow dots were the strains from Inner Mongolia and Yunnan Province, respectively.
